# Maternal and child factors associated with late neonatal bathing practices in Nigeria: evidence from a national survey

**DOI:** 10.1186/s12978-023-01676-y

**Published:** 2023-09-02

**Authors:** Francis Appiah, Kenneth Setorwu Adde, Kingsley Boakye, Justice Ofosu Darko Fenteng, Andrews Ohene Darteh, Tarif Salihu, Edward Kwabena Ameyaw, Patience Ansomah Ayerakwah

**Affiliations:** 1 Department of Population and Health, College of Humanities and Legal Studies, University of Cape Coast, Cape Coast, Central Region Ghana; 2 Berekum College of Education, Berekum, Bono Region Ghana; 3 School of Public Health, Kwame Nkrumah University of Science and Technology, Kumasi, Ashanti Region Ghana; 4 Department of Epidemiology and Biostatistics, School of Public Health, Kwame Nkrumah University of Science and Technology, Kumasi, Ashanti Region Ghana; 5 School of Public Health, Faculty of Health, University of Technology Sydney, Sydney, Australia; 6 L & E Research Consult Ltd, Wa, Upper West Region Ghana; 7 Department of Optometry, University of Cape Coast, Cape Coast, Central Region Ghana

**Keywords:** Late neonatal bathing, Maternal and child factors, Nigerian Demographic and Health Survey, Neonatal health, Reproductive health

## Abstract

**Background:**

Twohundred and seventy out of every thousand live births died in Nigeria in 2019. These deaths were attributable to infections, complications of preterm birth, and intrapartum-related conditions. The World Health Organization recommends withholding bathing of neonates until 24 h after birth or until their vital signs become stable to prevent hypothermia. Despite the link between neonatal bathing and thermal control, the subject is understudied in Nigeria. This study aimed at investigating the factors associated with late neonatal bathing practices in Nigeria.

**Methods:**

The study adopted a cross-sectional design and extracted data from the women’s file of the 2018 Nigerian Demographic and Health Survey. The unit of analysis was limited to 12,972 women who had complete data for the study. We applied chi-square test of independence to ascertain the association between the outcome variable and explanatory variables. At 95% confidence interval, two logistic regression models were built with Model I consisting of only maternal factors whilst Model II contained both maternal and child factors, and results were presented in adjusted odds ratio.

**Results:**

Descriptively, 12% (CI = 0.122–0.134) of the women bathed their neonates after 24 h of delivery. Inferentially, women with secondary/higher education [AOR = 1.30, CI = 1.05–1.61], the rich [AOR = 1.24, CI = 1.03–1.50], those with access to mass media [AOR = 131, CI = 1.15–1.50], women that professed other religions [AOR = 9.28, CI = 4.24–17.56], those who delivered in a health facility [AOR = 1.93, CI = 1.66–2.25], whose child was small in size at birth [AOR = 1.46, CI = 1.21–1.77] and delivered by caesarean section [AOR = 2.50, CI = 1.97–3.18] had higher odds of bathing their neonates 24 h after birth.

**Conclusions:**

The proportion of women who practised late neonatal bathing was generally low. To improve the practice of late neonatal bathing, much-concerted effort should be directed to women’s education and approaches to increasing receptivity of late neonatal bathing among pregnant women through the media. The Nigerian Ministry of Health should incorporate routine counselling on the risks of bathing newborns prematurely into antenatal and postnatal care services.

**Supplementary Information:**

The online version contains supplementary material available at 10.1186/s12978-023-01676-y.

## Background

Neonatal mortality rate (NMR) is a global health challenge. Globally, in 2019, 17 deaths per 1000 live births were reported among neonates in their first month of life, representing 6700 neonatal deaths every day [1]. Sub-regional disparities in NMR exist and the situation is more pronounced in sub-Saharan Africa (SSA) [1]. The SSA recorded 27 deaths out of every 1000 live births in 2019 whereas neonates born in the continent are 10 times more likely to die in their first month of life [1]. Nigeria is among the countries with the highest burden of NMR worldwide, which is 270 deaths per 1000 live births in 2019 [1]. These mortalities were due to inadequate care for neonates and are largely avoidable through low-cost interventions [2, 3], and these have made reducing neonatal mortalities difficult [4].

Generally, three major factors that account for neonatal deaths are infections, complications of preterm birth, and intrapartum-related neonatal deaths [5, 6]. Consequently, the World Health Organization (WHO) recommended a protocol for newborn care practices to improve the health outcomes of neonates. This included clean cord care, thermal protection, early and exclusive breastfeeding, delayed bathing, care for newborns with low-birth-weight, and management of newborns [7, 8]. The outlined protocol is sufficient practice necessary to keep the newborn’s temperature between 36.5 and 37.5 °C, which is much desired [6]. Additionally, to avoid hypothermia, the temperature must be kept warm just after birth. The reason is that neonates have a huge body surface area, thin skin, little insulating fat, and overworked thermoregulation mechanisms. As a result, newborns lose four times the amount of heat than adults do per unit of body weight [9, 10].

Neonatal bathing is traditionally practised globally to cleanse newborns of contamination by *vernix caseosa* and interrupt skin-to-skin contact [11]. The WHO and Save the Children International, however, indicate that newborns should not be bathed in the first 24 h, but should be delayed until their vital signs stabilize, as this will leave the remaining vernix caseosa intact and permit it to wear off with standard care and handling [12, 13]. Hypothermia which is related to early neonatal bathing can resort to life-threatening conditions such as low blood sugar, respiratory distress, irregular coagulation, jaundice, pulmonary bleeding, and an augmented danger of infection [10]. Furthermore, delaying bathing for at least 24 h allows newborn’s temperature to settle at 36.8 °C or higher and this has the potential to reduce hypothermia which is more likely to occur in the first hour after birth [14]. Also, the vernix caseosa, a protective fetal film that acts as a chemical and mechanical barrier in utero, can be kept intact, with the thickest coating developing between 36 and 38 weeks of gestation [13]. The advantages of leaving this coat are to protect newborns from infection, skin washing and moisturizing, and fortification of host defence proteins that are needed for innate immunity.

Despite the link between neonatal bathing and thermal control [14, 15], and child-related health [16–21], the subject is understudied in Nigeria. Hence, this study seeks to investigate the prevalence and factors associated with late neonatal bathing practices in Nigeria, utilizing a nationally representative survey dataset. Understanding these factors associated with delaying neonatal bathing is of public health importance. At least, it will be useful for the development of evidence-based interventions targeted at improving neonatal health outcomes [22].

## Methods

### Design and extraction of data

The study adopted a cross-sectional survey design. Data was extracted from the women’s file of 2018 Nigerian Demographic and Health Survey (2018 NDHS). The survey was implemented by the National Population Commission (NPC) whereas Inner-City Fund (ICF) provided technical assistance. The primary focus of the 2018 NDHS was to provide current statistics that reflect the basic demographic and health indicators for women, children, and men. Issues covered were fertility awareness, and use of family planning methods, breastfeeding practices, nutritional status of women and children, maternal and child health, adult and childhood mortality, women’s empowerment, domestic violence, female genital cutting, the prevalence of malaria, awareness and behaviour regarding HIV/AIDS and other sexually transmitted infections (STIs), disability, and other health-related issues such as smoking. This information was gathered using several questionnaires including the women’s questionnaire. The women’s questionnaire solicited information from all eligible women. They were asked questions covering their background characteristics, birth history and child mortality; knowledge, use, and source of family planning methods; antenatal, delivery, and postnatal care; vaccinations and childhood illnesses; breastfeeding and infant feeding practices and other health-related topics [23].

### Sample selection

**Fig. 1:**
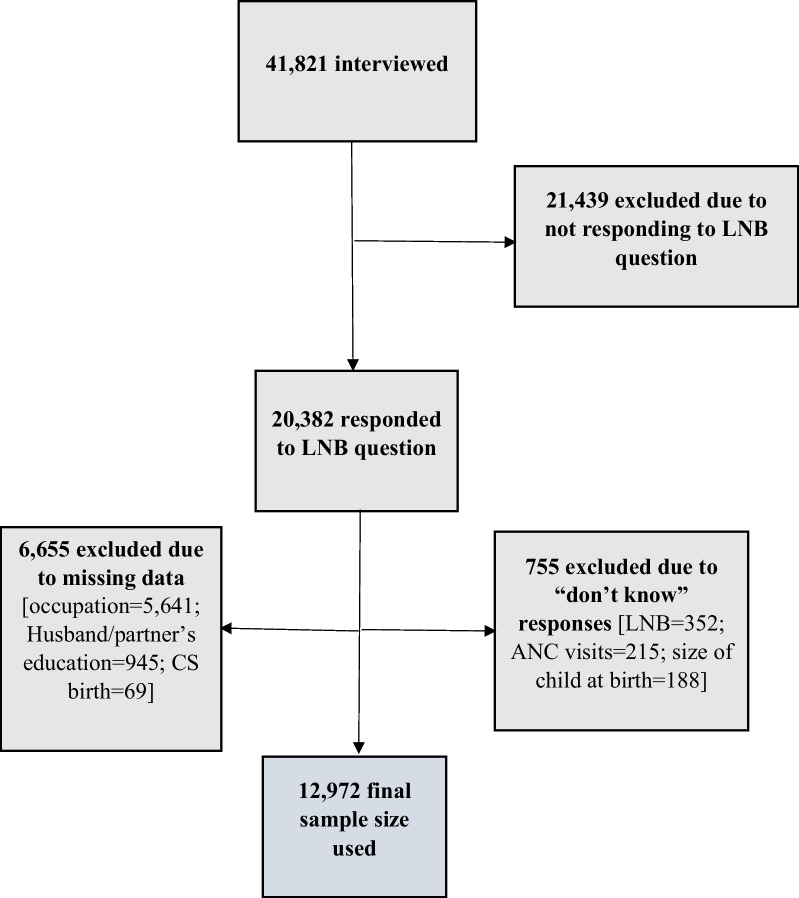
Sample size for the study

The 2018 NDHS used a stratified sampling approach to select eligible respondents. In the first stage, 1,400 enumeration areas (EAs) were selected with probability proportional to EA size. EA size was the number of households in the EA. A household listing operation was carried out in all selected EAs, and the resulting lists of households served as a sampling frame for the selection of households in the second stage. In the second stage, a fixed number of 30 households were selected in every cluster through equal probability systematic sampling, resulting in a total sample size of approximately 42,000 households. The interviewers conducted interviews only in the pre-selected households. To prevent bias, no replacements and no changes of the pre-selected households were allowed in the implementing stages. Due to the non-proportional allocation of the sample to the different states and the possible differences in response rates, sampling weights were calculated, added to the data file, and applied so that the results would be representative at the national level. In all, 42,121 women aged 15–49 years were identified for the study. However, a total of 41,821 women completed the survey and which translated to 99% response rate [23]. A total of 20,382 responded to the bathing practices question (i.e., LNB). Finally, our unit of analysis was limited to 12,972 women due to “missing data” and “don’t know responses” (see Fig. 1).

### Explanation of study variables

#### Outcome variable

In the 2018 NDHS survey, eligible women were specifically asked, “how long after birth was (NAME) bathed for the first time” and the responses were: (1) immediately; (2) hours; (3) days; and (4) don’t know. For specificity’s sake, “don’t know” responses were excluded from our analysis. After that, women that declared “immediately” and “hours (implied less than 24 h/a day)” were classified as “early neonatal bathing”. On the other hand, those that affirmed “days” were classified as “late neonatal bathing (LNB)”. We finally coded “early neonatal bathing” as “0” and “late neonatal bathing” as “1”. The outcome variable for the study was therefore “late neonatal bathing” conceptualized as delaying the bathing of newborns until 24 h after birth. The classification follows that of research in Lebanon [13] and by WHO [8].

#### Explanatory variables

Seventeen explanatory variables of theoretical importance were selected, comprising thirteen (13) maternal factors and four (4) child factors. The maternal factors are age, level of education, marital status, wealth status, level of employment, religion, residence, region, parity, access to mass media, health decision-making capacity, place of delivery, and antenatal care (ANC) visits. The child factors included the sex of the child, twin status, size of the child at birth, and delivery by caesarean section. Some of the variables were recoded for clarification purposes and to enhance easy comprehension by our readers. Education was recoded into ‘no education’, ‘primary’, and ‘secondary/higher’. Marital status recoded as ‘never married’, ‘married’, ‘cohabiting’, ‘widowed’, and ‘divorced’. Wealth status also recoded into ‘poor’, ‘middle’ and ‘rich’; level of employment recoded as ‘clerical’, ‘sales’, ‘services’, ‘manual’ and ‘agricultural’; the religion of affiliation recoded as ‘Christian’, ‘Muslim’, ‘Traditional’ and ‘others’. Considering the current fertility rate of Nigeria (which is 5.3 children per woman) [23], parity was recoded as ‘one birth’, ‘two births’, ‘three births’ and ‘four births’, and ‘five or more births’; health decision making also recoded into ‘no’ and yes. Access to mass media was computed from three different principal variables which are the frequency of reading newspaper/magazine, frequency of listening to the radio, and frequency of watching television which were asked during the 2018 NDHS. Each of these variables had three responses: ‘not at all’, ‘less than once a week’, and ‘at least once a week’. A composite variable was created whereby all ‘less than once a week’ and ‘at least once a week’ responses were classified as having access to mass media whilst ‘not at all’ was considered as not having access to mass media. Health decision-making was recoded into ‘alone’, ‘partner’, and ‘others’; place of delivery recoded as ‘home’, ‘health facility’ and ‘other’ and Antenatal Care (ANC) visits recoded as ‘less than eight visits’ and ‘eight or more visits’. Twin status was recoded as ‘single birth’ and ‘multiple births’ and the child’s size at childbirth recoded as ‘large’, ‘average’, and ‘small’.

### Statistical analysis

All the analyses were carried out using Stata statistical software version 14.0. First, descriptive computation of women that practised late neonatal bathing was done to describe the sampled general characteristics. At 5% alpha level, we conducted a chi-square test of independence to ascertain the association between our outcome variable and explanatory variables. The weighting factor inherent in the 2018 NDHS was applied to cater for sampling errors. Additionally, we checked for multicollinearity between our explanatory variables using variance inflation factor (VIF) and the results indicated no evidence of collinearity between them (Maximum VIF = 2.26; Minimum VIF = 1.01, Mean VIF = 1.48) (see Additional file 1: Appendix S1). At 95% confidence interval, we built two logistic regression models. In our first model (Model I), we computed the likelihood to practice late neonatal bathing according to maternal factors. We added child factors in Model II. Our results were presented as adjusted odds ratio (aOR).

### Ethical considerations

The study made use of a dataset extracted from the women’s file of the 2018 NDHS. As such, no ethical clearance was sought. However, permission to use the dataset was sought from the MeasureDHS platform and access was granted before we used the dataset. However, MeasureDHS anonymised the dataset before making it publicly available.

## Results

### Descriptive statistics for the study

Generally, less than one-third of women aged 15–49 bathed their neonates after 24 h of delivery (n = 1613, 12%; CI = 0.122–0.134) whilst a substantial fraction of them did otherwise (n = 11,359, 88%; CI = 0.866–0.878) (data not shown). Those that have completed secondary/higher education (40%), married (97%), the poor (41%), Clerical working class (57%), Muslims (56.30%%), reside in rural areas (59%), at parity five or more (40%) and have no access to mass media (59%) dominated. The majority of them delivered at home (53%), gave birth to a male child (51%), gave birth to an averagely weighted child (53%), and had a natural delivery (97%) (Table 1).

**Table 1:** Descriptive statistics for the study (*weighted N* = *12,972*)

Explanatory variables	Weighted (N)	Weighted (%)	Neonatal bathing practice		
			Early (%)	Late (%)	X^2^ (p-value)
Maternal factors					
Age					42.0212 (0.000)
15–19	426	3	91	9	
20–24	2016	16	90	10	
25–29	3388	26	87	13	
30–34	3136	24	85	15	
35–39	2392	18	87	13	
40–44	1146	9	87	13	
45–49	468	4	91	9	
Education					592.6987 (0.000)
No education	5182	40	95	5	
Primary	2121	16	88	12	
Secondary/higher	5669	44	80	20	
Marital status					0.0065 (0.936)
Married	12,520	97	87	13	
Cohabiting	451	3	87	13	
Wealth status					501.6894 (0.000)
Poor	5309	41	94	6	
Middle	2765	21	87	13	
Rich	4898	38	79	21	
Employment					86.6846 (0.000)
Professional/managerial	1080	8	79	21	
Clerical	7372	57	88	12	
Sales	713	5.80	90	10	
Services	1148	9	84	16	
Manual	2637	20	89	11	
Agricultural	22	0.20	91	9	
Religion					814.3554 (0.000)
Christian	5590	43	80	20	
Muslim	7302	56.30	94	6	
Traditional	58	0.50	90	10	
Other	22	0.20	20	80	
Place of residence					183.0611 (0.000)
Urban	5325	41	82	18	
Rural	7647	59	90	10	
Region					1.5e+03 (0.000)
North central	1976	15	92	8	
North east	2123	17	96	4	
North west	4037	31	95	5	
South east	1456	11	72	28	
South south	1261	10	61	39	
South west	2119	16	89	11	
Parity					59.5009 (0.000)
One birth	1684	13	84	16	
Two births	2348	18	86	14	
Three births	1965	15	85	15	
Four births	1770	14	88	12	
Five or more births	5205	40	90	10	
Access to mass media					405.5917 (0.000)
No	7703	59	92	8	
Yes	5269	41	80	20	
Health decision making					232.0294 (0.000)
Alone	1459	11	84	16	
Respondent/partner	4695	36	82	18	
Others	6818	53	92	8	
Place of delivery					447.0010 (0.000)
Home	6820	53	93	7	
Health facility	5888	45	81	19	
Other	264	2	78	22	
ANC visits					77.2358 (0.000)
< 8 visits	9941	77	89	11	
≥ 8 visits	3031	23	82	18	
Child factors					
Sex of the child					0.9728 (0.324)
Male	6614	51	87	13	
Female	6358	49	88	12	
Twin status					15.9537 (0.000)
Single birth	12,716	98	87	13	
Multiple births	256	2	79	21	
Size of child at birth					40.6352 (0.000)
Large	4303	33	90	10	
Average	6872	53	86	14	
Small	1797	14	88	12	
Delivery by caesarean section					219.5509 (0.000)
No	12,569	97	88	12	
Yes	403	3	63	37	

Computed from 2018 NDHS

From Table 1, LNB peaked among women aged 30–34 (15%) and those with secondary/higher education (20%). Thirteen percent of the married and the cohabiting practised LNB. LNB was highest among the rich (21%), women at professional/managerial rank (21%), women affiliated to other religion (80), urban residents (18%) and those in the South south region (39%). LNB was prevalent among women at parity one (16%), those who had access to mass media (20%), those that jointly make health decisions with partners (18%) and delivered using other facilities (22%). Women who made eight or more ANC visits (18%) and gave birth to a male child (13%) dominated LNB practice. LNB was remarkable among multiple births (21%), average size at birth (14%) and delivered by caesarean section (37%). Finally, from the chi-square test of independence, except for marital status [*X*^2^ = 0.0065, *p-value* = 0.936] and sex of the child [*X*^2^ = 0.9728, *p-value* = 0.324], the rest of the maternal and child factors were associated with LNB.

### Inferential statistics for the study

Table 2 is a hierarchical logistic regression for the study. Women with secondary/higher education had a higher likelihood to practice LNB compared with those with no education [AOR = 1.30, CI = 1.05–1.61]. Compared with the poor, the rich had higher odds to practice LNB [AOR = 1.24, CI = 1.03–1.50]. The likelihood to practice LNB decreased among women that are into sales relative to those engaged in the professional/managerial level [AOR = 0.71, CI = 0.52–0.97]. Relative to women affiliated with the Christian religion, the odds to practice LNB increased among women that professed other religions [AOR = 9.28, CI = 4.24–17.56]. Residents in the rural setting had lesser odds to practice LNB as compared to those in the urban areas [AOR = 0.83, CI = 0.74–0.96], just as among those who had eight or more ANC visits than their counterparts who had less than eight ANC visits [AOR = 0.69, CI = 0.60–0.80].

**Table 2:** Hierarchical logistic regression for the study

Explanatory variables	Model I		Model II	
	aOR	95% CI	aOR	95% CI
Maternal factors				
Age				
15–19	Ref.	1,1	Ref.	1,1
20–24	0.99	[0.66–1.47]	1.01	[0.67–1.51]
25–29	1.03	[0.69–1.53]	1.04	[0.69–1.55]
30–34	1.11	[0.74–1.67]	1.08	[0.72–1.63]
35–39	0.90	[0.59–1.38]	0.85	[0.56–1.31]
40–44	1.08	[0.69–1.69]	1.03	[0.66–1.62]
45–49	0.94	[0.56–1.57]	0.90	[0.53–1.51]
Education				
No education	Ref.	1,1	Ref.	1,1
Primary	1.18	[0.95–1.47]	1.20	[0.96–1.48]
Secondary/higher	1.29*	[1.04–1.60]	1.30*	[1.05–1.61]
Wealth status				
Poor	Ref.	1,1	Ref.	1,1
Middle	1.15	[0.96–1.37]	1.14	[0.95–1.36]
Rich	1.26*	[1.04–1.52]	1.24*	[1.03–1.50]
Employment				
Professional/managerial	Ref.	1,1	Ref.	1,1
Clerical	0.94	[0.78–1.13]	0.97	[0.80–1.17]
Sales	0.70*	[0.51–0.96]	0.71*	[0.52–0.97]
Services	0.84	[0.66–1.07]	0.87	[0.69–1.12]
Manual	0.91	[0.72–1.14]	0.93	[0.74–1.17]
Agricultural	0.66	[0.13–3.28]	0.59	[0.11–3.06]
Religion				
Christian	Ref.	1,1	Ref.	1,1
Muslim	0.85	[0.70–1.02]	0.84	[0.70–1.02]
Traditional	1.12	[0.45–2.76]	1.17	[0.50–2.92]
Other	9.28***	[4.57–18.87]	8.63***	[4.24–17.56]
Residence				
Urban	Ref.	1,1	Ref.	1,1
Rural	0.83**	[0.73–0.95]	0.84*	[0.74–0.96]
Region				
North central	Ref.	1,1	Ref.	1,1
North east	0.86	[0.67–1.12]	0.89	[0.69–1.15]
North west	1.20	[0.93–1.53]	1.22	[0.95–1.56]
South east	3.02***	[2.46–3.72]	3.09***	[2.51–3.81]
South south	5.99***	[4.86–7.37]	6.10***	[4.95–7.52]
South west	0.99	[0.80–1.24]	1.02	[0.82–1.28]
Parity				
One birth	Ref.	1,1	Ref.	1,1
Two births	0.75**	[0.61–0.92]	0.75**	[0.61–0.92]
Three births	0.88	[0.71–1.08]	0.89	[0.72–1.10]
Four births	0.76*	[0.60–0.95]	0.77*	[0.61–0.97]
Five or more births	0.87	[0.69–1.08]	0.91	[0.72–1.14]
Access to mass media				
No	Ref.	1,1	Ref.	1,1
Yes	1.30***	[1.14–1.48]	1.31***	[1.15–1.50]
Health decision making				
Alone	Ref.	1,1	Ref.	1,1
Respondent and partner	0.97	[0.82–1.16]	0.98	[0.82–1.17]
Others	0.91	[0.75–1.10]	0.93	[0.77–1.13]
Place of delivery				
Home	Ref.	1,1	Ref.	1,1
Health facility	2.04***	[1.76–2.37]	1.93***	[1.66–2.25]
Others	1.91***	[1.33–2.75]	1.95***	[1.36–2.81]
ANC visits				
< 8 visits	Ref.	1,1	Ref.	1,1
≥ 8 visits	0.71***	[0.62–0.82]	0.69***	[0.60–0.80]
Child factors				
Twin status				
Single birth			Ref.	1,1
Multiple births			1.69**	[1.21–2.36]
Size of child at birth				
Large			Ref.	1,1
Average			1.36***	[1.20–1.55]
Small			1.46***	[1.21–1.77]
Delivery by caesarean section				
No			Ref.	1,1
Yes			2.50***	[1.97–3.18]

Sources: NDHS 2018

*aOR* adjusted odds ratio, *CI* confidence interval in square brackets, *Ref* reference category

*p < 0.05, **p < 0.01, ***p < 0.001

Women in South south region had higher odds to practice LNB as compared to those in the North central region [AOR = 6.10, CI = 4.95–7.52]. The likelihood to practice LNB reduced among women at parity two compared with those at parity one [AOR = 0.75, CI = 0.61–0.92]. Those who had access to mass media had higher odds of LNB compared with those who had no access [AOR = 131, CI = 1.15–1.50]. Women that delivered using health facilities had a higher likelihood to practice LNB compared to those that delivered at home [AOR = 1.93, CI = 1.66–2.25]. It was evident that the odds to practice LNB increased among multiple births [AOR = 1.69, CI = 1.21–2.36], small size at birth [AOR = 1.46, CI = 1.21–1.77], and delivery by caesarean section [AOR = 2.50, CI = 1.97–3.18] compared with single births, large babies and vaginal delivery respectively (Table 2).

## Discussion

To preserve body temperature and reduce the risk of hypothermia, newborns should not be bathed until at least 24 h following delivery [24]. Newborns are frequently faced with health issues such as hypothermia shortly after delivery. As a result, the WHO recommended delaying newborn bathing until 24 h to reduce infant morbidity and mortality [10]. The key factors that were found to be significantly associated with late neonatal bathing were education, wealth status, employment, religion, place of residence, region, parity, access to mass media, place of delivery, twin status, size of child at birth, and delivery by caesarean section. In general, the study revealed that a little over one-tenth (12%) of women bathed their children within 24 h of birth. This study’s findings were lower than that of Saaka and co [25], who found that only about 23% of women bathed their newborns within 24 h of delivery in rural parts of Northern Ghana. Perhaps, the differences in the study population surveyed among Ghana and Nigeria could explain our observation.

In this present study, women with secondary or higher levels of education were more likely to practice LNB. This affirms the findings of previous studies. For instance, Tegene et al. [24] found that women with higher educational levels were more likely to practice LNB than women with low educational levels. Another study in Nepal found that women with higher levels of education were significantly more likely to engage in LNB [26]. The mother’s education was found to be strongly related to LNB in a study by Kaphle [27]. Similarly, research in Uganda and Ethiopia indicated that mothers with a high degree of education have a significant link with infant care practices such as LNB [28, 29]. Tegene et al. [24] found that maternal education was positively linked with LNB in their research. This could be because educated women were expected to have a high level of understanding regarding the importance of LNB practice.

In terms of wealth status, the study discovered that women who were rich had higher odds of practising LNB than poor women. When compared to impoverished women, rich women were more likely to engage in LNB. This result was in line with a study by Gul et al. [30], which found that a woman’s wealth status is directly related to her use of LNB. This was also in line with Chhetri et al. [31], who discovered that wealth status has a role in LNB practice. Similarly, Adegun et al. [32] found that wealth status was positively related to LNB in an Ibadan study. One probable explanation is that women with greater socioeconomic status have a greater educational level.

Regarding maternal employment, women in sales had a lower likelihood of practising LNB. This conclusion supported previous research conducted in Northern Ethiopia and Tigray by Berhe et al. [33] and Misgna et al. [5], which found that women who worked in professional/managerial level jobs were more likely to practice LNB than those who worked in sales. This could be attributed to the fact that professional/managerial work allows women to advance in their community, allowing them to access education, health care, decision-making, and financial independence. Women are more likely to practice LNB as a result of this.

Relative to women affiliated with the Christian religion, the odds to practice LNB increased among women that professed other religions. Perhaps, this could be attributed to the varied religious teachings Christians are exposed to. However, the cross-sectional nature of the study design did not permit exploring the reasons for this observation. The recent study discovered that LNB practice is strongly linked to place of residence. In comparison to their urban counterparts, women in rural areas were less likely to practice LNB. Possible reasons include a lack of maternal health services in rural areas compared to urban areas, as well as women in rural areas being expected to have lower maternal education than women in urban regions. This finding is also similar to Misgna et al. [5], who found that women who lived in rural areas were less likely to perform LNB than women who lived in urban areas, with those living in urban areas being roughly seven times more likely to perform LNB. Another study in Southern Ethiopia by Chichiabellu et al. [34] found that women in urban areas were more likely to perform LNB than women in rural areas.

LNB practice was also found to be highly associated with region. When compared to women in the North Central region, women in the South–South region were most likely to use LNB. Women in the South–South region were more likely than those in the North Central region to practice LNB. This could be attributed to regional differences in socio-demographic and economic status. The poor socio-economic condition of the North Central region may be due to poor literacy, which might lead to low unemployment and, as a result, low income, which explains the variation [24]. It is well known that northern women are devoted to their cultural beliefs and customs, as they would wish to bathe their newborns owing to the appearance of vernix and caseosa on their bodies, which they consider dirty.

Furthermore, the study discovered that a woman’s number of births was strongly linked to practise of LNB. When compared to women at parity one, women at parity two were less likely to use LNB. This result is consistent with a previous study by Bhatt et al. [35], which found that women who had more than one birth were less likely to use LNB. Alemu et al. [36] found that parity level was substantially correlated with LNB practice, which corroborated the current conclusion. In contrast to the current findings, Misgna et al. [5] found that women who had multiple babies were more likely to practice LNB than women who had one. Women who had more than one kid were more likely to practice LNB than women who only had one child, according to Welay et al. [10]. These disparities could be due to inequalities in the socioeconomic level of women and study participants.

Those who had access to the mass media were more likely to use LNB than women who did not. Women who learned about bathing time for their newborns from the media were more likely than their peers to undertake optimal newborn care, including LNB. This is due to the fact that the mass media informs women about the components and importance of appropriate newborn care, including LNB. As a result, women receive all of the required information to better grasp the benefits of LNB [17, 36].

The study also discovered that women who gave birth in a health institution were more likely to use LNB. The rural nature of the research zone, where negative cultural ideas and practices associated with LNB are ingrained, could be one possible cause. A study conducted in Zambia by Shamba et al. [37] indicated that the main reason for bathing newborns early was to clear away the blood/fluid/vernix that remained on the skin of the newborn baby. Ayiasi et al. [38] found that women who deliver in a health facility are more likely to use LNB than women who birth at home, which is consistent with the findings of the current study. Similarly, Iganus [39] found that birth in a health facility was a significant driver of LNB, with LNB being nearly universal for women who delivered in a health facility. Kumola [40], on the other hand, found no link between the place of birth and LNB. Furthermore, Baqui et al. [41] found that the place of delivery was not a factor of LNB in cross-sectional research done in India. However, women who had eight or more ANC visits were less likely to practice LNB. Having enough ANC visits presumably ought to expose expectant mothers to information on healthy practices such as LNB, therefore, this result is surprising. As such, a further study to explore this observation is worthy to be conducted. The current study found that the odds to practice LNB increased with multiple births, small neonate size at birth, and delivery by caesarean section compared to single births, large babies and vaginal delivery, respectively. These findings are consistent with a study in Malawi and Bangladesh where women who delivered by caesarean section were more likely to practice LNB when compared to women who delivered vaginally [42]. Similarly, Semanew et al. [17] discovered that women who gave birth via caesarean section were 43.8 per cent more likely to use LNB than those who gave birth vaginally. In contrast, Alemayehu and colleagues [43] found a link between the mode of birth and appropriate newborn care practices such as LNB, indicating that mothers who had a caesarean section were less likely to practice LNB.

## Strength and limitations

The study is unique because it is the first to investigate factors linked to late neonatal bathing in Nigeria. The study makes use of cross-sectional survey data, thus, the findings and conclusions are based on a nationally representative survey. The study also used a variety of data collection methodologies, with a relatively high response rate. However, there are certain limitations to the study. First, the study design prevents causal conclusions from being taken from the findings. Second, the women who were pooled are likely to have recollection and social desirability biases. The cross-sectional nature of the study design restricted the effort to unravel the reasons behind some of the observations.

## Conclusions

Late neonatal bathing is critical for reducing neonatal hypothermia and mortality while increasing newborn health. One of the most pressing public health issues affecting SSA countries, including Nigeria, is poor maternal and neonatal health outcomes. The number of women who practised LNB were found to be low. Secondary/higher education, wealth status, employment, religion, living in urban areas, region, parity, access to mass media, place of delivery, ANC visits, twin status, size of child at birth and delivery by caesarean section are the factors associated with LNB. To improve the practice of LNB, a significant amount of effort should be put into women’s education. Besides, the Ministry of Health should incorporate routine counselling on the risks of bathing newborns prematurely into ANC and postnatal care, as well as the provision of in-service training to natal attendants.

## Recommendation for practice and future research

Health professionals especially public health officials, should advocate, educate and strengthen all newly delivered mothers to practice late neonatal bathing since it will assist prevent neonatal hypothermia thereby helping to prevent neonatal mortality at large. Since the study is novel in Nigeria, future studies should investigate the contribution of late neonatal bathing to the reduction in neonatal mortality in Nigeria and elsewhere.

### Supplementary Information

**Additional file 1: Appendix S1.** Multicollinearity test results.

## Data Availability

The datasets generated and/or analysed during the current study are available in the Measure DHS repository at https://dhsprogram.com/data/dataset/Nigeria.

## Abbreviations

AOR: Adjusted odds ratio
ANC: Antenatal care
EAs: Enumeration areas
ICF: Inner-city fund
LNB: Late neonatal bathing
NMR: Neonatal mortality rate
NDHS: Nigerian Demographic and Health Survey
NPC: National Population Commission
PNC: Postnatal care
STIs: Sexually transmitted infections
SDG: Sustainable Development Goal
SSA: Sub-Saharan Africa
VIF: Variance inflation factor
WHO: World Health Organization
