# Supplementary material for: Maternal and child factors associated with late neonatal bathing practices in Nigeria: evidence from a national survey
Source: Reprod Health. 2023 Sep 2;20:131. doi: 10.1186/s12978-023-01676-y (PMC10474679; doi:10.1186/s12978-023-01676-y)
Supplement: Supplementary file 1 — Additional file 1: Appendix S1. Multicollinearity test results. [file 12978_2023_1676_MOESM1_ESM.docx]

**Appendix S1: Multicollinearity test results**

| Explanatory variable | VIF | 1/VIF |
| --- | --- | --- |
| Education | 2.26 | 0.442670 |
| Wealth | 2.12 | 0.471552 |
| Parity | 1.93 | 0.519290 |
| Age | 1.80 | 0.556349 |
| Religion | 1.57 | 0.637320 |
| Place of delivery | 1.54 | 0.650646 |
| Access to mass media | 1.53 | 0.655591 |
| Residence | 1.42 | 0.705842 |
| ANC visits | 1.32 | 0.759155 |
| Region | 1.28 | 0.782624 |
| Employment | 1.21 | 0.825073 |
| Health decision making | 1.19 | 0.842601 |
| Delivery by caesarean section | 1.05 | 0.951700 |
| Twin status | 1.02 | 0.982644 |
| Size of the child at birth | 1.01 | 0.986286 |
| Mean VIF | 1.48 |  |
